# Effect of low-dose gamma irradiation on seed-borne transmission of tomato brown rugose fruit virus in tomato

**DOI:** 10.1016/j.jgeb.2025.100644

**Published:** 2026-01-05

**Authors:** Kimia Tokhmechi, Abozar Ghorbani, Davoud Koolivand, Mahsa Rostami, Nahid Hajiloo

**Affiliations:** a Department of Plant Protection, Faculty of Agriculture, University of Zanjan, Zanjan, Iran; b Nuclear Agriculture Research School, Nuclear Science and Technology Research Institute (NSTRI), Karaj, Iran; c Radiation Application Research School, Nuclear Science and Technology Research Institute, Karaj, Iran

**Keywords:** Gamma irradiation, RT-qPCR, Seed disinfection, Sodium hypochlorite, Tomato brown rugose fruit virus (ToBRFV)

## Abstract

Tomato brown rugose fruit virus (ToBRFV), a highly virulent tobamovirus, poses a major threat to global tomato production by overcoming host resistance and traditional control measures. This study evaluates the efficacy of low-dose gamma irradiation (10, 15, and 20 Gy) in reducing ToBRFV contamination in tomato seeds. Contaminated seeds were irradiated and assessed for germination rate, chlorophyll content, stem diameter, and viral accumulation with RT-qPCR. The potential synergistic effect of combining 15 Gy gamma irradiation with 2.5 % sodium hypochlorite (NaOCl) was also investigated. Results revealed that 15 Gy significantly improved germination, enhanced chlorophyll levels, and increased stem thickness, while substantially reducing viral replication. In contrast, 20 Gy had detrimental effects on both plant growth and viral suppression. The combination of 15 Gy and NaOCl further decreased viral accumulation, though at the cost of reduced germination rates. Applying 15 Gy confers dual benefits, including effective seed disinfection and improved host resistance. It shows strong potential for use in integrated tomato disease management in greenhouse and field conditions.

## 1 Introduction

Tomato (*Solanum lycopersicum* L.) is one of the most widely cultivated vegetable crops globally, valued for both its nutritional content and economic importance. However, tomato production is increasingly threatened by emerging plant pathogens, particularly *Tobamoviruses*, known for their environmental stability and efficient mechanical transmission.^1^ Among these, Tomato brown rugose fruit virus (ToBRFV), a novel tobamovirus first identified in the Middle East, has caused severe outbreaks in various countries^2^, overcoming resistance in commercial cultivars and resulting in substantial yield losses.^3^ The virus primarily spreads through infected seeds, contaminated tools, human activity, and insect pollinators, such as *Bombus terrestris*^4^. Although seed transmission rates appear low (0.8–2.8 %), they are sufficient to initiate infection in greenhouse and field environments.^5^ ToBRFV contamination has been detected on 100 % of seeds derived from infected fruits, even though the virus is generally confined to the seed coat^6^. The global trade and movement of commercial seed lots exacerbate this risk, facilitating long-distance dissemination of the virus.^7^

To date, the management of ToBRFV largely relies on phytosanitary practices, such as crop rotation, destruction of infected plant residues, and seed disinfection using chemical agents like sodium hypochlorite (NaOCl). While NaOCl is effective in removing surface-borne viral particles, it has limited efficacy against internal seed infections and does not stimulate systemic plant defenses.^8^ Moreover, the overuse of chemical disinfectants raises concerns about phytotoxicity, environmental impact, and potential for resistance development.

An alternative approach gaining attention is the use of low-dose gamma irradiation, particularly using cobalt-60 ({\,}^60Co) sources. Gamma irradiation has long been applied in agriculture for purposes such as induced mutation breeding, insect sterilization, and food preservation.^9^ At sublethal doses (5–30 Gy), gamma rays can elicit beneficial physiological responses in plants, including enhanced growth, increased antioxidant activity, and activation of systemic acquired resistance (SAR)^10^. These effects are mediated through the generation of reactive oxygen species (ROS), which act as signaling molecules to upregulate defense genes such as superoxide dismutase (SOD) and phenylalanine ammonia-lyase (PAL).^11^ Previous studies have demonstrated the antiviral potential of gamma irradiation against other *Tobamoviruses*. For instance, Jeong et al. (2017) reported successful inactivation of Tobacco mosaic virus (TMV) virions at high doses, whereas Mardani-Mehrabad et al.^10^ showed reduced Bean common mosaic virus (BCMV) titers in beans at lower doses through the induction of host defense responses. Nevertheless, there remains a critical gap in evaluating the dual utility of gamma irradiation for both ToBRFV disinfection and the induction of antiviral resistance mechanisms in tomato.

This study addresses this gap by assessing the effects of low-dose gamma irradiation (1–20 Gy) on ToBRFV-infected tomato seeds. The findings aim to contribute to the development of a safe, effective, and sustainable strategy for the integrated management of ToBRFV in tomato production systems.

## 2 Materials and methods

### 2.1 Sample collection and molecular detection of ToBRFV

To assess potential ToBRFV infection, symptomatic samples—including entire plants with root systems, rhizosphere soil, and seeds of the commercial tomato cultivar 'SV 3725TH'—were collected from greenhouses in Isfahan Province using sterile techniques. Samples were immediately placed in temperature-controlled containers and transported to the laboratory under cold-chain conditions, then stored at − 80 °C to maintain sample integrity for subsequent molecular and virological analyses.

Detection of ToBRFV was carried out via reverse transcription polymerase chain reaction (RT-PCR) using a Bio-Rad PTC-1148 thermal cycler. Total RNA was extracted from symptomatic tomato fruits using TRIzol™ reagent (Thermo Fisher Scientific), following the manufacturer’s instructions. The extracted RNA was treated with DNase (Thermo Scientific) for first-strand cDNA synthesis using the cDNA synthesis kit (Pishgam, Iran). For virus-specific amplification, primer pairs described by^12^, ^13^ were used in combination with Taq DNA Polymerase Master Mix RED (Denmark). Amplification products were separated by 1 % agarose gel electrophoresis and visualized using ethidium bromide staining. All procedures were conducted in strict accordance with established reagent concentrations and thermal cycling conditions.

### 2.2 Greenhouse trial 1- evaluation of gamma irradiation effects on ToBRFV-infected tomato seeds

In the first greenhouse trial, ToBRFV-infected tomato seeds of the commercial cultivar 'SV 3725TH' were treated with three different doses of gamma irradiation (10, 15, and 20 Gy) using a Theratron-780 Cobalt-60 ({\,}^60Co) source at a dose rate of 77.35 mGy/min. Untreated seeds served as the control group. Dose corrections for radioactive decay were applied to ensure accurate exposure. Seeds were placed in specialized Petri dishes within the gamma-ray field to guarantee uniform irradiation.

The gamma irradiation doses (10, 15, and 20 Gy) were selected based on prior studies demonstrating that low-dose gamma irradiation (5–30 Gy) can enhance seed germination, activate systemic acquired resistance, and suppress viral pathogens in solanaceous crops without causing significant phytotoxicity.^11^, ^14^ Preliminary germination assays in our laboratory further confirmed that doses below 10 Gy had minimal impact on ToBRFV suppression, while doses above 20 Gy reduced seed viability. Thus, the 10–20 Gy range was chosen to evaluate the trade-off between antiviral efficacy and seedling vigor within a biologically relevant dose–response window.

Following treatment, seeds were sown in separate trays and grown under controlled greenhouse conditions with daytime temperatures maintained at 25–30 °C and nighttime temperatures at 16–20 °C, 60–70 % relative humidity, 250 μmol/m^2^/s light intensity, and a 16 h light/8h dark photoperiod. Irrigation was performed every other day. The experiment was designed as a completely randomized design (CRD) with four treatments corresponding and 40 replications to the irradiation doses and a control. Once seedlings reached the four-leaf stage, virus presence was assessed by Reverse Transcription quantitative PCR (RT-qPCR) to determine the effect of irradiation on ToBRFV infection. Seed germination rates were recorded throughout the growth period to evaluate the impact of gamma irradiation on seed viability. Additionally, stem diameter was measured below the last vegetative leaf using a caliper, and chlorophyll content was assessed using a Hansatech CL-01 chlorophyll meter.

### 2.3 Relative quantification of ToBRFV viral accumulation using optimized RT-qPCR with specific primers

To detect the virus by qPCR, specific primers targeting the ToBRFV coat protein gene were used.^15^ The tubulin gene was employed as an internal control for normalization purposes (Table 1). To quantify viral accumulation across treatments—including control (CG) and gamma-irradiated groups at 10, 15, and 20 Gy (G10–G20)—real-time quantitative PCR (RT-qPCR) assays were conducted. It is important to note that RNA extraction and cDNA synthesis were performed as described in earlier stages to confirm viral infection in plant samples.

**Table 1:** Nucleotide sequences of primers used for amplification of ToBRFV and the internal control gene (tubulin).

**Gene**	**Primer**	**Sequence (5′ → 3′)**
**ToBRFV**	ToB5520F	GTAAGGCTTGCAAAATTTCGTTCG
**ToBRFV**	ToB5598R	CTTTGGTTTTTGTCTGGTTTCGG
**Tubulin**	Tubulin For	GAATATCAACAATACCAGGATGC
**Tubulin**	Tubulin Rev	AGGATTGGTATTGATCATCAGCA

RT-qPCR reactions were optimized using MASTER SYBR Green 2× (Ampliqon Co., Denmark). Each 12.5 μL reaction mixture contained 6.25 μL SYBR Green master mix, 0.25 μL of each primer (10 pmol), 2 μL of cDNA template, and nuclease-free water to reach the final volume. Thermal cycling conditions consisted of an initial denaturation at 95 °C for 15 min, followed by 40 cycles of denaturation at 95 °C for 30 s, annealing at 58 °C for 30 s, and extension at 72 °C for 30 s, concluding with a final extension step at 72 °C for 1 min. The experiment was performed with three biological replicates and two technical replicates for each sample. Threshold cycle (Ct) values were recorded, and transcript abundance was analyzed in Microsoft Excel by normalizing Ct values to the Tubulin gene. The relative expression of target genes was determined using the 2^-ΔΔCT method.^16^

### 2.4 Statistical analysis

All experimental data were analyzed using Python 3 in the Jupyter Notebook environment, with core libraries including Pandas, NumPy, SciPy, Stats, Matplotlib, Seaborn, and Statsmodels.

Datasets were first imported from Excel files and cleaned by converting column headers to string format. Outlier detection and removal were performed using interquartile range (IQR) and Z-score methods to ensure statistical robustness. For all measured traits, including germination percentage, stem diameter, and chlorophyll content, a one-way analysis of variance (ANOVA) was conducted to assess differences among treatment groups. When significant effects were observed (p < 0.05), Tukey’s Honest Significant Difference (HSD) test was used for post-hoc pairwise comparisons. Data visualization was performed using seaborn for trend and comparison plots. Also, a high number of biological replicates (n = 40) were used for germination assays to ensure statistical power for this critical production parameter, while n = 3 biological replicates with two technical replicates each were used for RT-qPCR, consistent with standard practices in virological quantification.

### 2.5 Greenhouse trial II- combined disinfection of ToBRFV-infected seeds using sodium hypochlorite and gamma irradiation

In the second phase of greenhouse trials, the effectiveness of combined chemical and physical seed disinfection methods was evaluated using sodium hypochlorite and gamma irradiation. A total of four treatment groups were established to assess individual and synergistic effects: (1) treatment with 2.5 % sodium hypochlorite alone (H), (2) treatment with 15 Gy gamma irradiation alone (G), (3) combined treatment of sodium hypochlorite followed by gamma irradiation (GH), and (4) untreated control (C) (with 40 replicates).

Seed disinfection began with immersion of seeds in 2.5 % sodium hypochlorite solution at room temperature for 15 min. Post-treatment, seeds were thoroughly rinsed three times with distilled water, each rinse lasting five minutes, to remove residual disinfectant. Treated seeds were then air-dried at room temperature for one week, as described by Ling et al.^17^ For the GH treatment group, dried seeds were subsequently exposed to 15 Gy of gamma irradiation. Following treatment, all seeds were sown into separate seedling trays and grown under controlled greenhouse conditions identical to those of the first experiment. Germination percentage, stem diameter, and chlorophyll content were recorded. In addition, RT-qPCR was performed to evaluate viral accumulation across treatment groups. Also, statistical analyses were performed similarly to Trial I.

## 3 Results & discussion

### 3.1 Molecular confirmation of ToBRFV infection by RT-PCR

**Fig. 1:**
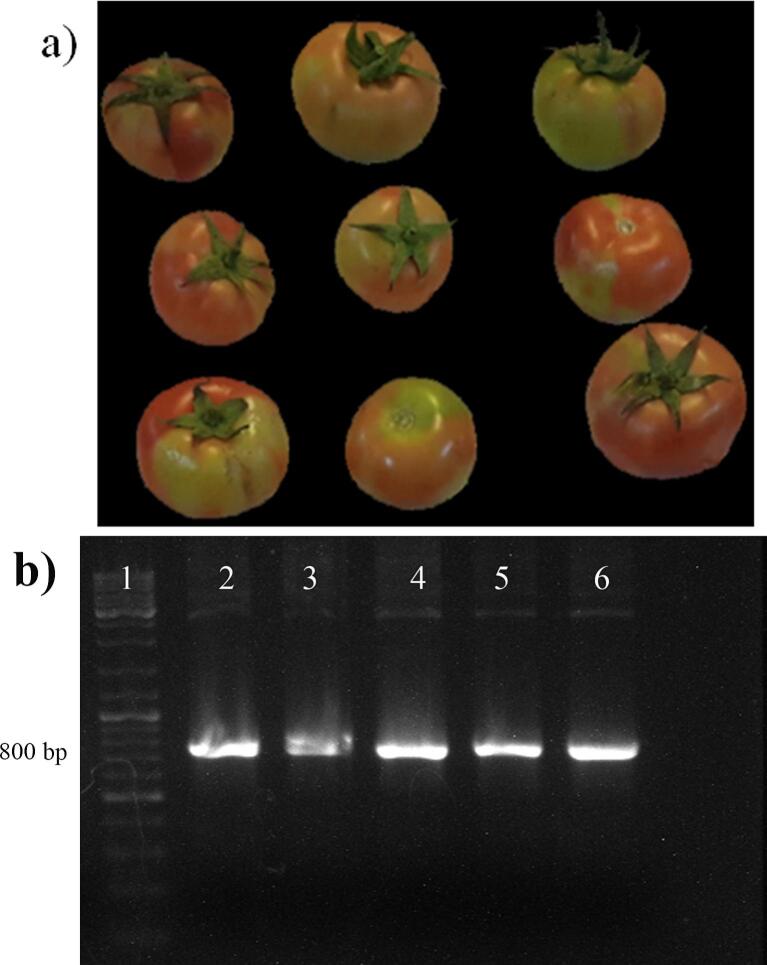
Symptomatic presentation (a) and molecular detection (b) of Tomato brown rugose fruit virus (ToBRFV). Lane 1: DNA ladder (2800 DM, SMOBIO, Taiwan). Lanes 2–5: samples; Lane 6: ToBRFV-positive sample.

The RT-PCR analysis confirmed the presence of ToBRFV in symptomatic samples. Amplification was performed using virus-specific primers targeting the envelope protein gene, yielding a distinct amplicon of 823 base pairs. The appearance of this expected band in all tested samples, as visualized by agarose gel electrophoresis (Fig. 1), provided molecular evidence of ToBRFV infection and validated the selection of plant material for downstream experiments.

### 3.2 First greenhouse trial

#### 3.2.1 Plant growth and physiological traits

##### 3.2.1.1 Impact of gamma irradiation on germination of ToBRFV-infected tomato seeds

**Fig. 2:**
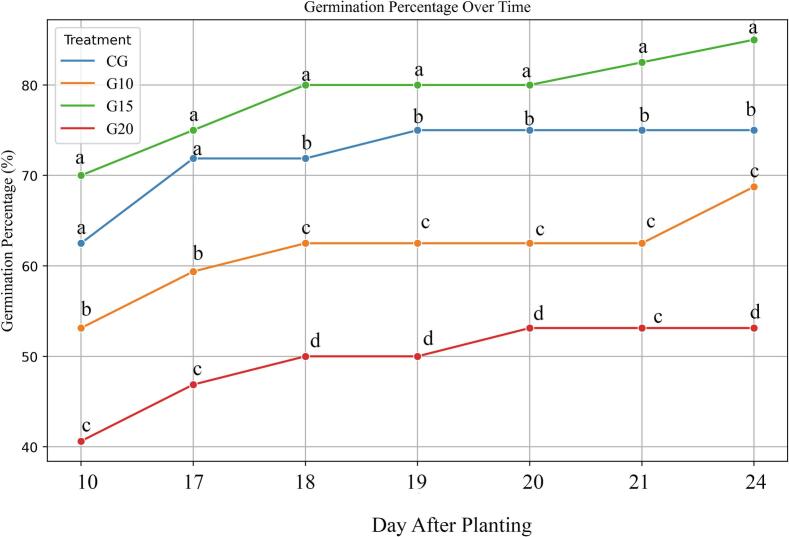
Effect of gamma irradiation on germination percentage of ToBRFV-infected tomato seeds over 14 days. CG – control; G10 – 10 Gy Gamma; G15 – 15 Gy Gamma; G20 – 20 Gy Gamma. Error bars represent ± standard deviation (n = 40 biological replicates). Statistical significance was determined by one-way ANOVA followed by Tukey’s HSD test at p < 0.05.

In the initial screening, tomato seeds were exposed to various low-dose gamma irradiation levels (10, 15, and 20 Gy) and evaluated for their germination performance under controlled conditions. Treated seeds were sown in seedling trays under standard greenhouse conditions, and germination progress was recorded over 14 days. Interestingly, seeds exposed to 15 Gy (G15) exhibited the highest germination percentage throughout the experiment, outperforming even the control (Fig. 2). This suggests that 15 Gy may exert a stimulatory effect on seed germination. No ToBRFV-like symptoms were observed in any seedlings up to the four-leaf stage. At this point, samples were collected to confirm the presence or absence of viral accumulation.

Numerous studies have demonstrated the influence of gamma irradiation on seed germination and early plant development. The germination and growth responses of plants to gamma irradiation are primarily influenced by factors such as radiation dose, exposure duration, and the specific plant species involved.^18^, ^19^ For instance, Verma et al.^20^ reported enhanced *Cuminum cyminum L* germination at 100 Gy but reduced it at higher doses. Hussain et al.^21^ identified the effective range for sunflower as 0.5–5 KR, while Beyaz et al.^22^ recommended 100–150 Gy for grass pea (*Lathyrus sativus*), and Salomón Díaz et al.^14^ found 20 Gy to be optimal for Solanum tuberosum L. Similarly, Aref et al.^23^ reported improved germination in Datura innoxia at 5 Gy, though higher doses were inhibitory. Lower doses of gamma irradiation promote plant growth by inducing direct genomic modifications or regulating cellular processes, including hormonal signaling, enhancing enzymatic activity, boosting antioxidant capacities, and modifying cell membrane structures, among others.^18^

In the present study, our first greenhouse trial showed that irradiation at 15 Gy (G15) led to a notable increase in germination percentage compared to the control, with no visible morphological abnormalities or ToBRFV symptoms. This stimulatory effect is likely due to enhanced enzymatic activity, increased nucleic acid and protein synthesis, and the activation of hormonal and signaling pathways—all processes previously associated with low to moderate doses of gamma irradiation.^11^ However, as the dose increased beyond 15 Gy, a gradual decline in germination percentage was observed. This inhibitory trend may be attributed to chromosomal aberrations or disruptions in cell division, particularly at the G2/M phase of the cell cycle, leading to reduced viability and growth. High doses are also associated with DNA damage, oxidative stress, and impaired metabolic functions.^11^ The enhanced germination response at 15 Gy supports the hypothesis that specific doses of gamma irradiation can serve as effective pre-sowing treatments to improve seed performance.

##### 3.2.1.2 Impact of gamma irradiation on chlorophyll content and stem diameter of ToBRFV-infected tomato plants

**Fig. 3:**
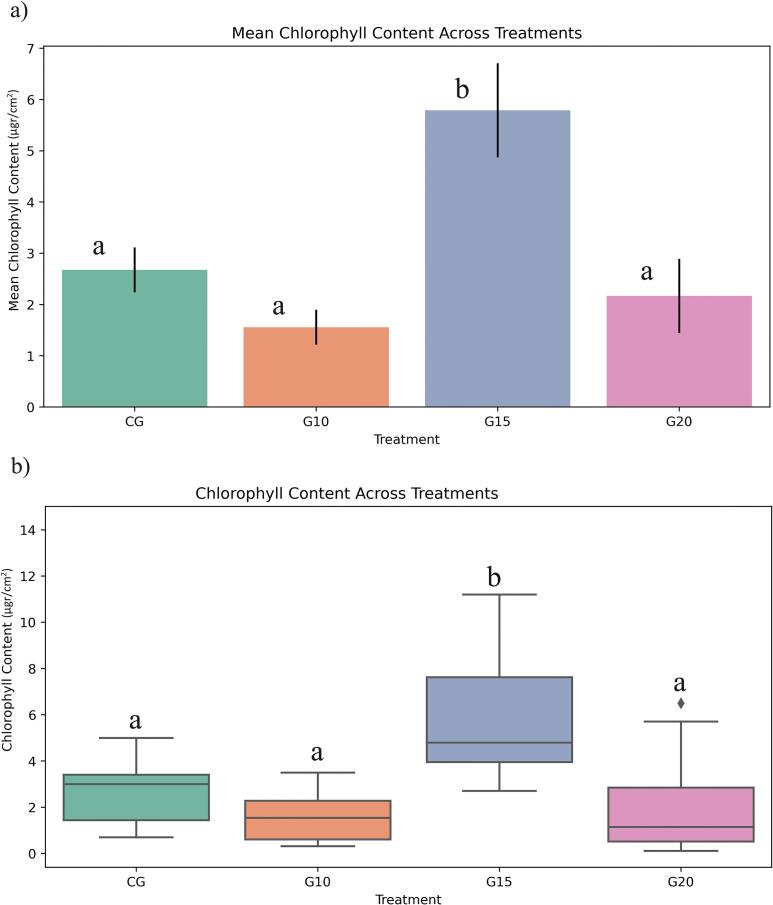
Effect of gamma irradiation (10, 15, 20 Gy) and control plants (CG) on chlorophyll content (µg cm^−2^) in ToBRFV-infected seedlings. Error bars represent ± standard deviation (n = 40 biological replicates). Statistical differences among treatments were determined by one-way ANOVA followed by Tukey’s HSD test at the level of p < 0.05. CG: Control, G10: 10 Gy Gamma, G15: 15 Gy Gamma, G20: 20 Gy Gamma.

The analysis of chlorophyll content indicated that plants treated with 15 Gy of gamma irradiation exhibited a significant increase in chlorophyll levels compared to the control group. In contrast, plants treated with 10 Gy and 20 Gy showed no significant differences in chlorophyll content relative to the control. The G15 treatment demonstrated a statistically significant increase in chlorophyll compared to both G10 and G20, suggesting that gamma irradiation influences chlorophyll accumulation (Fig. 3).

Regarding chlorophyll content, studies have reported varied effects of gamma irradiation. Found that in Arabidopsis thaliana, chlorophyll levels remained stable up to 60 Gy. Furthermore, a recent study on soybeans reported that 12 Gy irradiation enhanced chlorophyll *a*, chlorophyll *b*, and carotenoid levels compared to non-irradiated seeds.^24^ The results of this study align with previous reports indicating that low doses of gamma irradiation positively affect plant growth and yield in hybrid tomato cultivars^25^. Also, Low doses of gamma rays resulted in the highest concentrations of chlorophyll *a*, chlorophyll *b*, and carotenoids in red radish leaves.^26^ This increase in photosynthetic pigments may be attributed to beneficial mutations induced by low-dose irradiation, leading to structural modifications in cells and enhanced physiological processes such as thylakoid membrane expansion and improved photosynthetic efficiency. These changes ultimately promote pigment accumulation, which influences leaf coloration.^27^ The significant increase in chlorophyll content at 15 Gy suggests that this dose optimally stimulates pigment accumulation, likely by enhancing photosynthetic efficiency.

**Fig. 4:**
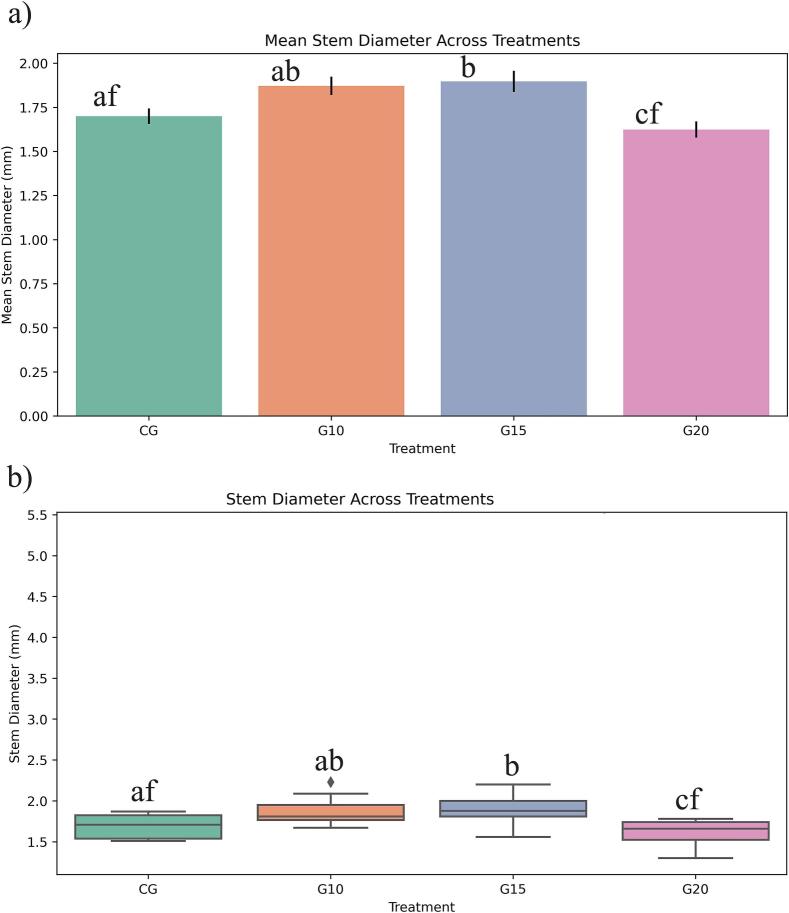
Effect of gamma irradiation (10, 15, 20 Gy) and control plants (CG) on stem diameter (mm) in ToBRFV-infected seedlings. Error bars represent ± standard deviation (n = 40 biological replicates). Statistical differences among treatments were determined by one-way ANOVA followed by Tukey’s HSD test at the level of p < 0.05. CG: Control, G10: 10 Gy Gamma, G15: 15 Gy Gamma, G20: 20 Gy Gamma.

Stem diameter analysis showed that plants treated with 15 Gy had the widest diameter distribution, with significant differences compared to both the control and G20 samples, but not with G10. The application of 10 Gy and 15 Gy appeared to enhance stem diameter, whereas increasing the irradiation dose to 20 Gy resulted in a reduction in stem diameter (Fig. 4).

Studies on Helianthus tuberosus showed that 5 Gy irradiation enhanced plant height, branch number, and shoot biomass^28^. From a physiological perspective, low doses of gamma irradiation can stimulate cell division in meristematic tissues, thereby enhancing vegetative growth characteristics^18^. The observed increase in plant height under low-dose treatments may be attributed to the promotion of vital cellular processes, including the stimulation of nucleic acid synthesis^29^. Furthermore, gamma irradiation at lower doses may enhance the activity of antioxidant systems and contribute to a favorable balance of endogenous hormones, both of which play critical roles in supporting plant growth and development.^11^ The increase in stem diameter at 10 and 15 Gy suggests that low-dose gamma irradiation has a positive influence on vegetative growth, likely through enhanced cell division and hormonal balance. In contrast, the decline at 20 Gy highlights the threshold beyond which irradiation becomes inhibitory to growth processes.

#### 3.2.2 Virological outcomes

##### 3.2.2.1 Relative quantification of ToBRFV viral accumulation in gamma irradiation-treated tomato plants

**Fig. 5:**
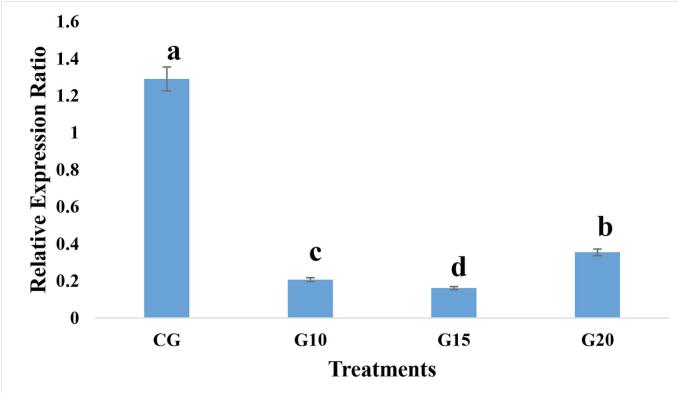
Relative ToBRFV accumulation determined by RT-qPCR (2^−^ΔΔCT method) in leaves of infected tomato plants treated with gamma irradiation (10, 15, 20 Gy) and control (CG). Values represent means ± standard deviation (n = 3 biological replicates, each with 2 technical replicates). Statistical significance was assessed using one-way ANOVA and Tukey’s HSD test (p < 0.05). Data are plotted on 2^(−ΔΔCT) values. CG: Control, G10: 10 Gy Gamma, G15: 15 Gy Gamma, G20: 20 Gy Gamma.

The RT-qPCR analysis, using a specific primer pair targeting the envelope protein gene of the ToBRFV, along with the internal control gene (*Tubulin*), revealed a significant increase in cycle threshold (CT) values in treated plants compared to control plants. This indicates a reduction in virus proliferation across all treatment doses (Fig. 5).

The results indicated that plants treated with 15 Gy exhibited the lowest level of viral accumulation. In contrast, treatment with 20 Gy increased viral accumulation, likely due to embryonic damage caused by the higher irradiation dose.^30^, ^31^ SAR is a form of long-term immunity in plants that is mediated through salicylic acid signaling and the activation of defense-related genes such as PR-1. Exposure to 15 Gy gamma irradiation may induce the controlled generation of ROS, which enhances salicylic acid signaling and activates SAR. In contrast, exposure to 20 Gy may cause excessive oxidative stress or potential damage to the embryo, thereby disrupting these pathways and impairing the induction of resistance.^10^, ^11^, ^32^ Thus, the balance between ROS signaling and oxidative damage determines whether gamma irradiation strengthens or weakens plant defense responses. A previous study examined the application of gamma irradiation for the inactivation of *Cucumber green mottle mosaic virus* (CGMMV) in Nicotiana tabacum. Their findings revealed a dose-dependent reduction in CGMMV infection, with complete viral inactivation achieved at doses exceeding 40 kGy. Electron microscopy analysis further confirmed that gamma irradiation disrupted the virion structure, leading to the degradation of viral proteins and genomic RNA.^32^ Based on these observations, it was anticipated that higher gamma doses would correlate with reduced viral accumulation. However, in the present study, the 20 Gy treatment unexpectedly resulted in increased viral accumulation. This phenomenon is likely due to irradiation-induced damage to plant embryos, where irradiation directly affects cellular macromolecules (such as nucleotides, ribonucleotides, and, to a lesser extent, proteins). Additionally, irradiation can indirectly damage these macromolecules by generating a significant flux of free oxygen radicals, particularly hydroxyl radicals and hydrogen peroxide. This disruption impairs growth indicators, including chlorophyll content and stem diameter, thereby preventing the effective induction of resistance.^30^, ^31^ As a result, the few remaining viral particles were able to replicate rapidly, leading to a higher overall viral accumulation. Consequently, 15 Gy was identified as the optimal irradiation dose for subsequent experiments (Fig. 5). Additionally, lower gamma doses were employed in this study to minimize the risk of mutation and growth impairment, which are commonly associated with higher irradiation levels.^33^

In the current study, all gamma-treated seeds exhibited reduced viral accumulation, but increasing the dose to 20 Gy negatively affected chlorophyll content, seedling diameter, and viral control. The 15 Gy dose was identified as the most effective, likely due to gamma-induced stress triggering plant defense mechanisms. This phenomenon aligns with findings from Mardani et al. (2020), who examined the impact of low-dose gamma irradiation (10, 20, and 30 Gy) alone and in combination with salicylic acid and indirect heat on *Bean common mosaic virus* (BCMV) contamination. Their results demonstrated significant improvements in growth indices in treated plants. Additionally, gamma irradiation increased the activity of antioxidant enzymes, protein density in infected and healthy seedlings, and reduced the concentration of BCMV-related proteins. The combination of gamma irradiation with salicylic acid or heat treatment further enhanced these effects, leading to greater resistance against BCMV. Similarly, Jeong et al. (2017) investigated the application of gamma irradiation for *Tobacco Mosaic Virus* (TMV) inactivation. Their findings showed a dose-dependent reduction in TMV infection, with complete inactivation occurring at doses above 40 kGy. Electron microscopy revealed that increased irradiation disrupted the virion structure and degraded viral proteins, ultimately leading to TMV inactivation. Although high-dose gamma irradiation was primarily applied to purified viral samples, the study suggested that combining irradiation with heat or environmentally friendly agents could reduce the required dose while effectively inactivating plant viruses. Further research explored the antibacterial effects of gamma irradiation on *Pseudomonas syringae*, the causative agent of bacterial spot disease in tomatoes. The results demonstrated that 200 Gy completely inactivated bacterial cells *in vitro*. Examination of irradiated bacterial cells revealed cytoplasmic disruption, membrane integrity loss, and genomic DNA fragmentation, with similar effects observed at lower doses^34^. Overall, excessive free radicals generated by high irradiation doses can lead to oxidative stress, impairing plant growth. Increasing gamma irradiation levels have been linked to reductions in fresh and dry weight, as well as root and stem length.^35^ However, at optimal low doses, gamma irradiation can induce stress responses that enhance plant resistance mechanisms. In this study, the 15 Gy dose emerged as the most effective, balancing viral suppression with improved growth characteristics, whereas 20 Gy led to adverse effects, likely due to irradiation-induced damage to plant viability.

### 3.3 Second greenhouse trial

#### 3.3.1 Plant growth and physiological traits

##### 3.3.1.1 Effect of gamma irradiation and sodium hypochlorite on germination of ToBRFV-infected seeds

**Fig. 6:**
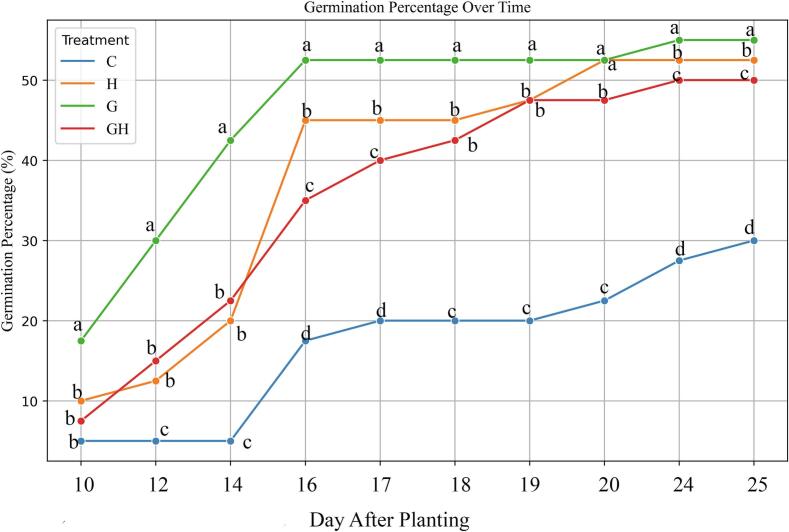
Effect of gamma irradiation and sodium hypochlorite on the germination percentage of ToBRFV-infected tomato seeds over 20 days. Treatments: C: Control, G: 15 Gy Gamma, H: Sodium Hypochlorite, GH: Sodium Hypochlorite + 15 Gy Gamma. Error bars represent ± standard deviation (n = 40 biological replicates). Statistical differences among treatments were determined by one-way ANOVA followed by Tukey’s HSD test (p < 0.05).

In this phase of the study, the optimal gamma irradiation dose (15 Gy) was assessed in combination with sodium hypochlorite. The germination rate and percentage in treated tomato plants indicated that the highest values were observed in the gamma irradiation (G) treatment. The germination rate and percentage were significantly higher in the G treatment compared to the control. However, plants treated with both gamma irradiation and sodium hypochlorite exhibited lower germination rates and percentages compared to those treated with gamma irradiation alone. The lowest germination rate and percentage were observed in the control plants, suggesting that the 15 Gy dose of gamma irradiation provided a more effective germination stimulus than sodium hypochlorite. Additionally, throughout the 20-day growth period, the germination percentage in the 15 Gy treatment consistently remained higher than in the other treatments (Fig. 6).

In this phase of the study, the optimal gamma irradiation dose (15 Gy) was evaluated in combination with sodium hypochlorite treatment. The results indicated that tomato seeds exposed solely to gamma irradiation (15 Gy) demonstrated the highest germination rate and percentage, with statistically significant improvements over the control group. These findings align with previous studies, which report that low-dose gamma irradiation can enhance seed germination and seedling vigor by inducing physiological and biochemical changes that stimulate growth^36^. However, seeds treated with both gamma irradiation and sodium hypochlorite exhibited reduced germination performance compared to those treated with gamma irradiation alone. While sodium hypochlorite is commonly used for seed surface sterilization and can moderately enhance germination,^37^ its combination with ionizing radiation may lead to plant stress or damage to essential biomolecules, thereby limiting its synergistic effect. The observed variation in the germination percentage range between the two experimental phases can be attributed to differences in seasonal and photoperiodic conditions during cultivation; despite this variation, the current results still fully support and validate the outcomes of the initial germination assay. Throughout the 20-day growth period, the 15 Gy treatment consistently maintained the highest germination percentage among all treatments. Furthermore, comparisons across both experimental phases confirmed that 15 Gy was the most effective dose, consistently promoting superior germination relative to other doses (10 Gy, 20 Gy) and the control. These findings underscore the role of 15 Gy gamma irradiation as a potent germination stimulant, while also suggesting that its combination with chemical disinfectants like sodium hypochlorite may be counterproductive.

##### 3.3.1.2 Impact of gamma irradiation and sodium hypochlorite on chlorophyll content and stem diameter of ToBRFV-infected tomato plants

**Fig. 7:**
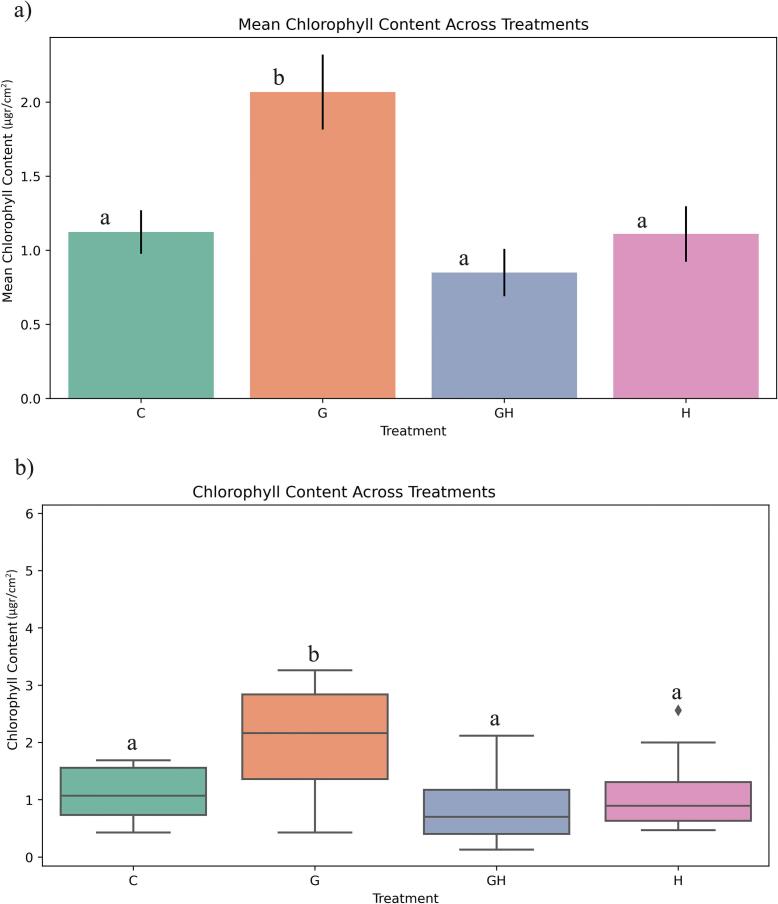
Effect of gamma irradiation and sodium hypochlorite on chlorophyll content (µg cm^−2^) in ToBRFV-infected seedlings. C: Control, G: 15 Gy Gamma, H: Sodium Hypochlorite, GH: Sodium Hypochlorite + 15 Gy Gamma. Error bars represent ± standard deviation (n = 40 biological replicates). Statistical differences among treatments were determined by one-way ANOVA followed by Tukey’s HSD test at the level of p < 0.05.

The comparative analysis of mean chlorophyll content and data distribution indicated a significant increase in chlorophyll levels in plants treated with gamma irradiation compared to the control, GH, and H samples. However, the combination of gamma irradiation and sodium hypochlorite did not exhibit a significant impact on chlorophyll content, as no notable difference was observed between these treatments (Fig. 7).

**Fig. 8:**
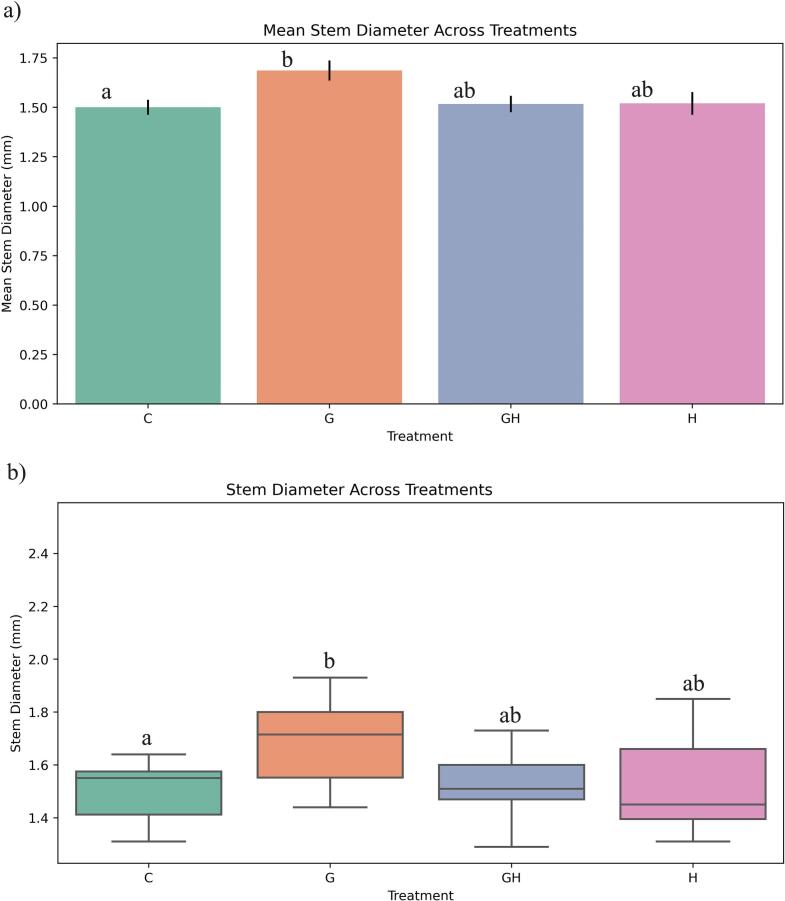
Effect of gamma irradiation and sodium hypochlorite on stem diameter (mm) in ToBRFV-infected seedlings. C: Control, G: 15 Gy Gamma, H: Sodium Hypochlorite, GH: Sodium Hypochlorite + 15 Gy Gamma. Error bars represent ± standard deviation (n = 40 biological replicates). Statistical differences among treatments were determined by one-way ANOVA followed by Tukey’s HSD test at the level of p < 0.05.

The analysis of mean stem diameter and data distribution showed that only the gamma irradiation (G) treatment resulted in a significant increase compared to the control. In contrast, the combination of gamma irradiation and sodium hypochlorite did not significantly affect stem diameter, as no substantial difference was detected (Fig. 8).

The analysis revealed that gamma irradiation at 15  Gy significantly enhanced chlorophyll content in ToBRFV-infected tomato plants compared to the control, GH, and H groups, confirming a positive physiological response. Gamma rays at low doses stimulate photosynthetic pigment accumulation by modulating electron transport proteins, chlorophyll, and antioxidant enzymes.^38^ In contrast, the combination of gamma irradiation with sodium hypochlorite did not yield additional benefits in chlorophyll levels. Stem diameter analysis mirrored these findings; only the G treatment exhibited a statistically significant increase compared to the control, indicating improved vascular development and turgor pressure, potentially due to radiation-enhanced metabolic activity. The combined treatment showed no effect on stem thickness, suggesting that sodium hypochlorite neither synergizes nor impairs gamma-induced structural enhancements. Collectively, data from both study phases confirm that 15  Gy gamma irradiation alone fosters notable improvements in both chlorophyll content and stem diameter under ToBRFV infection, while the inclusion of sodium hypochlorite does not further enhance these physiological traits.

#### 3.3.2 Virological outcomes

##### 3.3.2.1 Relative quantification of ToBRFV viral accumulation in gamma irradiation and sodium hypochlorite-treated tomato plants

**Fig. 9:**
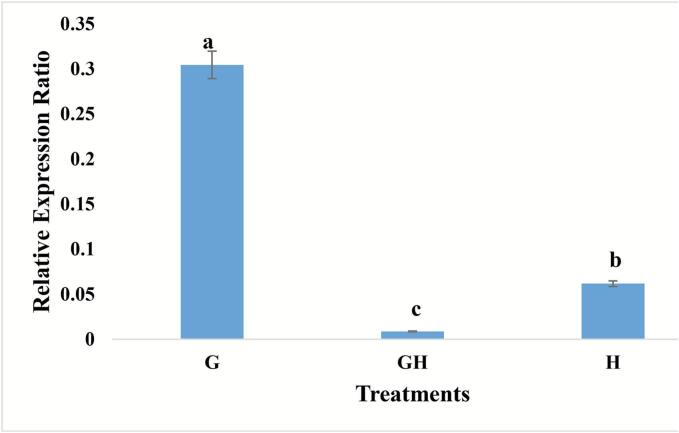
Relative ToBRFV accumulation in leaves of infected tomato plants treated with 15 Gy gamma irradiation (G), 15 Gy + sodium hypochlorite (GH), and sodium hypochlorite alone (H).

The results of RT-qPCR indicate the positive effects of sodium hypochlorite and gamma irradiation treatments. Specifically, the GH treatment exhibited an increased CT value, signifying reduced viral proliferation. According to Fig. 9, the GH treatment had the lowest viral accumulation compared to the H and G treatments.

Values represent means ± standard deviation (n = 3 biological replicates, each with 2 technical replicates). Statistical significance was determined using one-way ANOVA and Tukey’s HSD test (p < 0.05). Data are plotted on 2^(−ΔΔCT) values.

The combination of 15 Gy gamma irradiation and sodium hypochlorite resulted in the most significant reduction in viral accumulation. In a prior study on seed disinfection for ToBRFV, the most effective virus control was achieved with a 0.25 % sodium hypochlorite solution^17^. The findings of the present study further indicate that incorporating gamma irradiation enhances virus control, likely due to gamma irradiation acting as a low-dose stressor that induces the activation of antiviral defense genes.^31^ At low doses (5–30 Gy), gamma irradiation can act as a resistant inducer that triggers beneficial physiological and defensive responses in plants^10^. One of the key outcomes of such exposure is the increased production of ROS, which serve as crucial signaling molecules in plant defense. ROS activate signaling cascades such as the MAPK pathway and stimulate the synthesis of salicylic acid, ultimately leading to the induction of SAR. During this process, key defense-related genes are upregulated, strengthening the plant’s immune system against ToBRFV infections.^11^ However, despite improved viral control, germination percentage, germination rate, and chlorophyll content remained higher in plants treated solely with gamma irradiation. These results align with the findings from the first phase of the experiment.

## 4 Conclusion

The emergence of ToBRFV as a global agricultural threat necessitates innovative, sustainable strategies to mitigate its impact on tomato production. This study demonstrates that low-dose gamma irradiation (15 Gy) effectively reduces ToBRFV contamination in seeds while enhancing plant growth and systemic resistance. The optimal dose of 15 Gy significantly improved germination rates, chlorophyll content, and stem diameter compared to untreated controls, suggesting its role in stimulating physiological and biochemical pathways critical for plant vigor. Notably, this dose reduced viral replication, likely by triggering antioxidant responses and defense-related gene expression, which prime plants for resistance. Conversely, higher doses (e.g., 20 Gy) impaired growth and viral control, underscoring the importance of balancing efficacy with phytotoxicity. The synergistic application of 15 Gy gamma irradiation with 2.5 % sodium hypochlorite further suppressed viral accumulation, highlighting its potential for integrated seed disinfection protocols. However, this combination yielded lower germination rates compared to irradiation alone, indicating that chemical treatments may introduce additional stress. Although the results are promising, the controlled conditions limit their applicability. Field trials are needed to confirm effectiveness, and more research is required to understand the molecular mechanisms behind gamma-induced resistance. While gamma irradiation shows strong promise for ToBRFV management in tomato seeds, its integration into commercial seed production requires consideration of logistical, economic, and regulatory factors. Gamma irradiation facilities, typically using^60Co sources, require higher initial capital investment than conventional chemical disinfection methods such as sodium hypochlorite baths, but offer long-term advantages including residue-free treatment, consistent efficacy, and reduced environmental impact. Operational costs are moderate, with treatment times for low-dose applications (e.g., 15 Gy) usually ranging from minutes to under an hour, making them easily compatible with existing seed processing workflows. However, strict radiation safety protocols– such as shielded irradiation chambers, personnel training, and regulatory licensing– are mandatory under national and international standards, including IAEA guidelines. Given its non-thermal, non-chemical nature and compatibility with organic and integrated seed health programmes, low-dose gamma irradiation could be positioned as a premium seed treatment option, particularly for high-value greenhouse tomato cultivars where ToBRFV poses a critical risk.

## CRediT authorship contribution statement

**Kimia Tokhmechi:** Writing – original draft, Validation, Software, Data curation. **Abozar Ghorbani:** Writing – review & editing, Supervision, Software, Investigation, Conceptualization. **Davoud Koolivand:** Writing – review & editing, Supervision, Formal analysis. **Mahsa Rostami:** Writing – review & editing, Validation, Software, Data curation. **Nahid Hajiloo:** Writing – review & editing, Methodology.

## Declaration of competing interest

The authors declare that they have no known competing financial interests or personal relationships that could have appeared to influence the work reported in this paper.
