# Supplementary figures and images for: Effect of low-dose gamma irradiation on seed-borne transmission of tomato brown rugose fruit virus in tomato
Source: J Genet Eng Biotechnol. 2026 Jan 5;24(1):100644. doi: 10.1016/j.jgeb.2025.100644 (PMC12810537; doi:10.1016/j.jgeb.2025.100644)

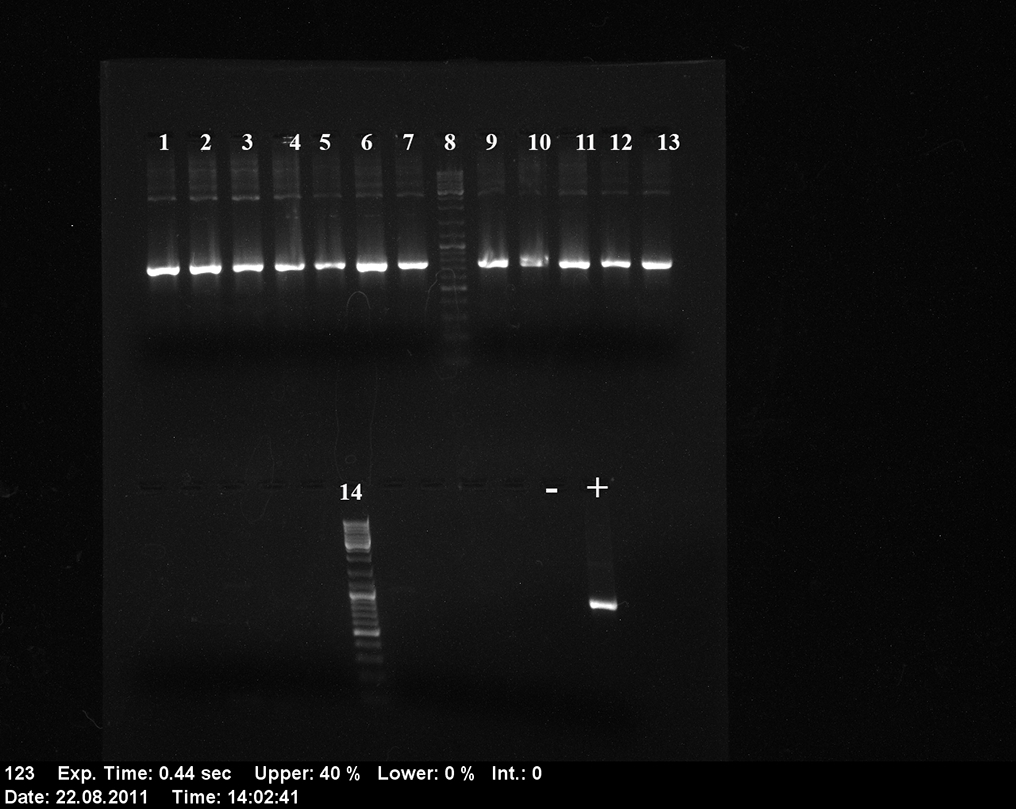

Supplement: Supplementary Figure 1 [file mmc1.jpg]
